# hnRNP A2B1 as a promising therapeutic target for radiomodulatory drug development: evidence from computational and experimental studies

**DOI:** 10.3389/fphar.2025.1704625

**Published:** 2026-02-25

**Authors:** N. S. Lubinets, I. S. Tonkii, V. A. Sazonova, K. Dutta, A. E. Putintseva, O. O. Volkova, E. V. Skorb, P. Zun, A. Ruzov, V. Yu. Kravtsov, S. Shityakov

**Affiliations:** 1 Department of Chemoinformatics, ITMO University, Saint-Petersburg, Russia; 2 Department of Faculty Surgery, Saint Petersburg State Pediatric Medical University, Saint Petersburg, Russia; 3 St. Petersburg National Research Academic University of the Russian Academy of Sciences named after Z.I. Alferov, Saint-Petersburg, Russia; 4 Institute of Bioengineering, Research Center of Biotechnology of the Russian Academy of Sciences, Moscow, Russia; 5 Sector of Personalised Biomedical Technologies, Saint-Petersburg Research Center of the Russian Academy of Sciences, Saint-Petersburg, Russia

**Keywords:** hesperidin, heterogeneous nuclear ribonucleoproteins, hnRNP A2B1, molecular docking, radiomodulating drugs, psoralidin, radiomodulators

## Abstract

Radiation modulation is critical due to the growing risks of radiation exposure in medical, occupational, and environmental settings. This study explored the potential of heterogeneous nuclear ribonucleoproteins (hnRNPs), particularly hnRNP A2B1, as therapeutic targets for radiomodulatory drugs. *In vitro* experiments on human endothelial cells exposed to various X-ray doses revealed a dose-dependent increase in hnRNP A2B1 expression, with the histochemical score increasing significantly from 105 to 280 at 8.0 Gy. Using in silico molecular docking, 33 radiomodulatory agents were evaluated for their binding affinities with hnRNP A2B1. Notable ligands, such as hesperidin and psoralidin, demonstrated strong binding affinities (ΔG_tot_ values: −17.2 and −17.9 kcal⋅mol^-1^, respectively). Finally, molecular dynamics simulations confirmed that psoralidin had the highest affinity for hnRNP A2B1 using implicit solvation models. Overall, this study revealed that hnRNP A2B1 is vital for cellular radiomodulation and is a promising target for radiomodulatory drugs that could have radioprotective or radiosensitizing effects.

## Graphical Abstract

**Figure:**
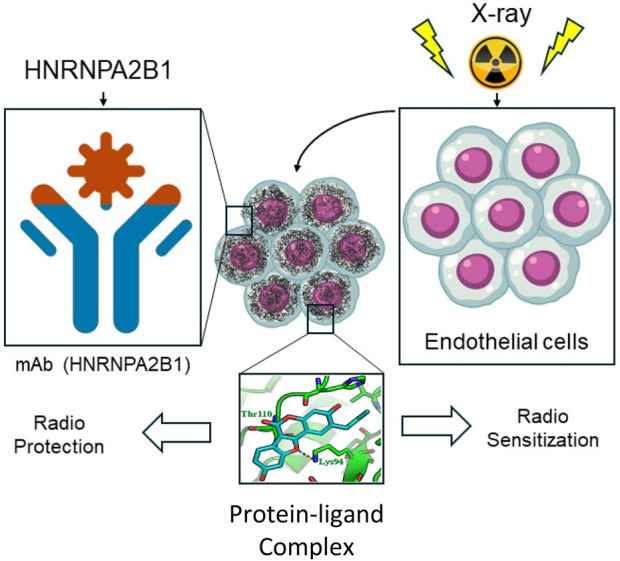

## 1 Introduction

The changing landscape of medical diagnostics and therapeutic interventions requires ongoing innovation in radiation shielding advanced materials to protect the safety of both healthcare workers and patients. Traditionally, lead aprons are the preferred choice for X-ray radiation protection. However, the continuous issues linked with their weight and toxicity have inspired researchers to work tirelessly toward X-ray protection (Ogul et al., 2024). Radiotherapy (RT) is main treatment for many cancer types (Haque et al., 2023; Mali, 2024; Peng et al., 2023; Vinod and Hau, 2020; Chandra et al., 2021; Zhang et al., 2022; Chen and Kuo, 2017; Gong et al., 2021; Rallis et al., 2021; Chen et al., 2020; Petroni et al., 2022; Pointer et al., 2022; Qian and Schoenfeld, 2021; Haussmann et al., 2020; Díaz-Gavela et al., 2021). However, maximizing radiation damage to tumor tissue while minimizing adverse effects on healthy tissue remains a significant problem. Adverse effects are manifested mainly through the production of free radicals that accelerate DNA damage. Radiosensitizers are a class of medicines that accelerate damage to tumor tissue. Numerous techniques have been developed to create radiosensitizers that are both highly efficient and have low toxicity, including small molecules, macromolecules, and nanomaterials (Wang et al., 2018). Conversely, radiomodifiers offer protection to affected tissues. Radiomodifiers fall into one of two categories: (a) radioprotectors, which shield molecules and tissues from radiation-induced direct and indirect damage, or (b) radiomitigators, which reduce and aid in the healing of damage (Montoro, 2023). Extensive research on radiomodulators explores their molecular structure, therapeutic potential, metabolic role, and mode of action (Nair et al., 2001). The development of radiomodulating medications, which shield the human body from radiation, began in the mid-20th century. Today, the production of highly effective radiomodulating drugs and approved natural medicines is a reality due to advancements in science and technology, including artificial intelligence. Among these advancements, the molecular docking approach stands out for its ability to detect and select radiomodulatory agents. Currently, heterogeneous nuclear ribonucleoproteins (hnRNPs) are among the most promising therapeutic targets (Moding et al., 2013; Tchurikov et al., 2013). Studies on hnRNPs and their crucial roles in genomic instability and repair offer entirely new avenues for radiation treatment (Sharifi-Rad et al., 2020). Specifically, hnRNP A2B1 is considered a target for novel medications, such as radiomodulators. In addition to being an oncogene that controls tumor suppressors and other oncogenes, the hnRNP A2B1 protein is a cancer biomarker and may be targeted for the treatment of various forms of cancer. Notably, the 28 amino acids in the C-terminus of hnRNP A2B1 (GRD3) exhibit greater binding affinity than the full-length protein does (Sharifi-Rad et al., 2020). Research indicates that hnRNP A2B1 is critical for cell survival.

Concurrently and in parallel with our project, studies are also being conducted (Xu et al., 2023; Wang et al., 2023) that report new data on the induction of YTHDF2 protein expression in malignant bone marrow cells with myelodysplastic syndrome. It turns out that YTHDF2 is a factor of radioresistance in malignant neoplasms during their radiotherapy. YTHDF2 is currently already being considered as a drug target for inhibition to enhance radiosensitivity during radiotherapy of malignant neoplasms. However, the question remains unanswered as to how the HNRNPA2B1 protein itself is expressed in this context and whether its expression in cells depends on their radiation dose. The HNRNPA2B1 and YTHDF2 proteins are functionally closely interconnected and influence each other within the same protein interactome. In light of the above, studying the expression of HNRNPA2B1 after irradiation has become a priority to directly establish its role in radioresistance.

In this context, the goal of this work was to perform theoretical *in silico* modeling via a molecular docking approach to investigate the interactions of the hnRNP A2B1 protein with known radiomodulators and natural radiomodulator agents and evaluate their binding affinities. Furthermore, hnRNP A2B1 may be evaluated as a new and potentially important therapeutic target for radiomodulating drugs, as these inhibitors or ligand-activated proteins can modulate radioresistance in human somatic cells. In this context, the goal of this work was to perform theoretical in silico modeling via a molecular docking approach to investigate the interactions of the hnRNP A2B1 protein with known radiomodulators and natural radiomodulator agents and evaluate their binding affinities.

## 2 Materials and methods

### 2.1 Tissue incubation of endothelial biopsies and irradiation

Biomaterials (biopsies and histological sections) for the conducted studies were obtained from one of the clinics in St. Petersburg. Participation in the study was based on the informed consent of the participants. The study’s compliance with international ethical standards, as outlined in the World Medical Association’s Helsinki Declaration “Recommendations for Doctors Involved in Biomedical Research Involving Human Subjects,” was confirmed by the decision of the Local Ethics Committee at SPbSPMU, protocol No. 20/03 dated 22.12.2022.

Under sterile conditions, the endothelial layer was separated, and fragments were divided into smaller pieces of approximately 5 mm^3^, which were then placed in sterile culture flasks with culture medium. The culture medium consisted of 35% Eagle’s MEM, 35% Hanks’ balanced salt solution, and 25% fetal bovine serum (BioloT LLC, Russia), with the remaining 5% comprising additives, including 6 g/L glucose, 0.5 IU/mL insulin, and 100 μg/mL gentamicin. Endothelial tissue samples in flasks were irradiated using the RUM-17 X-ray therapeutic unit, with an X-ray tube voltage of 180 kV, current of 10 mA, focal distance of 50 cm, and dose rate of 0.39 Gy/min. The doses of X-ray irradiation used were 0.5, 1, 2, 4, and 8 Gy. Control samples were placed in the irradiation chamber with the X-ray tube off for the same duration as the irradiated samples. The irradiation was conducted at the Scientific and Clinical Toxicology Center named after Academician S.N. Golikov, FMBA of Russia, under a scientific cooperation agreement with ITMO National Research University’s Research Center for Informatics, dated 22 January 2021.

#### 2.1.1 Human participants and demographics

Human endothelial biopsies were obtained from 88 adult patients undergoing elective surgery for symptomatic chronic hemorrhoidal disease. The mean age was 45.4 ± 10.7 years (range 23–74), with 48 males and 40 females. Clinical grading indicated grade II (n = 64), grade III (n = 22), and grade IV (n = 2) hemorrhoidal disease. According to the American Society of Anesthesiologists (ASA) physical status classification, 10 patients were ASA I, 75 ASA II, and 3 ASA III. Smoking status was recorded (19 smokers, 69 non-smokers). Exclusion criteria included active malignancy, prior pelvic radiotherapy or chemotherapy within 6–12 months, uncontrolled diabetes with advanced angiopathy, systemic vasculitis, decompensated cardiovascular or renal disease, and severe autoimmune disorders with vascular involvement. All participants provided written informed consent. The study was approved by the Local Ethics Committee at SPbSPMU (Protocol No. 20/03, 22.12.2022).

### 2.2 Immunocytochemical analysis of the hnRNP A2B1 protein

Immunocytochemical staining of smears to detect the expression of the hnRNP A2B1 protein was performed simultaneously on all glass slides. To visualize hnRNP A2B1 protein expression in smears of human endothelial tissue after in vitro culture (14 and 24 h postirradiation) at various doses, monoclonal mouse antibodies against hnRNP A2B1 (M01A, clones 1G12--6C5; Abnova Headquarters, Taiwan) were used. Additionally, a three-step Novolink Polymer Detection System (Leica Biosystems, United Kingdom) was utilized in the study. The results of the immunocytochemical reaction were evaluated via the standard method widely accepted in clinical practice, the S_H_ method. The S_H_ calculation system included assessing the intensity of immunocytochemical staining on a three-point scale and the percentage (%) of stained cells. It represents the sum of the products of the percentages reflecting the proportion of cells with different staining intensities by the score corresponding to the intensity of the reaction. The staining intensities were as follows: 0 - no staining, 1 - weak staining, 2 - moderate staining, and 3 - strong staining. The calculation formula is as follows:

$$S_{H}=\sum \left(i\times P\left(i\right)\right)$$

where the histochemical score (S_H_) represents the histoscore, *i* is the staining intensity ranging from 0 to 3, and *P*(*i*) is the percentage of cells stained with different intensities.

Therefore, the maximum value of the S_H_ should be 300. One hundred cells in various fields of view were counted. The S_H_ results are interpreted as follows: from 0 to 10, negative; from 10 to 100, weakly positive; and from 100 to 300, positive.

Endothelial identity was confirmed by characteristic cobblestone morphology and, in parallel preparations, by CD31 immunostaining (Novolink Polymer Detection System). For hnRNP A2B1 scoring, only cells with typical endothelial morphology were counted; obvious non-endothelial contaminants (e.g., spindle-shaped stromal cells, epithelial cells) were excluded. The biopsy material was intrinsically enriched in endothelial cells (≈70–80% of the cytogram) as established in our patented method (RU 2022108179A). While flow cytometric purity assessment was not performed, the consistent dose-dependent increase in hnRNP A2B1 expression across all donors suggests the observed effects are endothelial-specific.

For the positive control in hnRNP A2B1 immunohistochemical staining, we used sections from a renal carcinoma patient known to overexpress hnRNP A2B1 (Li et al., 2024). These sections were stained concurrently with hemorrhoidal samples using identical antibodies, visualization systems, and protocols as part of routine diagnostic procedures. Renal carcinoma was selected due to its established hnRNP A2B1 hyperexpression, making it an effective positive control (Supplementary Figure S1A).

### 2.3 Structure-based computational screening

The structures of the hnRNP A2B1 protein were downloaded from the PDB (Protein Data Bank), PDB identifier: 5HO4. To calculate the electrostatic potential of the protein‒RNA complex, we used the adaptive Poisson‒Boltzmann solver (APBS) algorithm through the PyMOL plugin. Ligand structures were obtained from the PubChem database. The Avogadro program was used to optimize the geometry of the ligand structures. The binding sites were predicted via the CASTp v.3.0 algorithm. The AutoDock Vina algorithm was used to perform molecular docking of ligands to ribonucleoprotein binding sites. The grid cell volume for hnRNP A2B1 was set to 50 × 50 × 50 Å with a step size of 0.375 Å. PyMOL software was used to visualize the resulting protein–ligand complexes. The Gibbs energy (ΔG) was used as the affinity parameter. After studying the literature on this topic, 33 radiomodulatory agents (Supplementary Table S1) with different action spectra were selected as ligands for docking (Vasin et al., 2010; Vasin, 2021; Nai et al., 2022; Sikorski et al., 2023). Among them, natural compounds that may have radiomodulatory activity are considered (Nai et al., 2022; Sikorski et al., 2023).

### 2.4 Molecular dynamics simulations

All-atom molecular dynamics (MD) simulations were conducted via the AMBER 18 package, which employs the FF99SB and general Amber force fields. The systems were solvated with TIP3P water models and neutralized via Na+ and Cl− ions via the tLEaP input script provided by AmberTools. Long-range electrostatic interactions were handled via the particle‒mesh Ewald method (Essmann et al., 1995), whereas the SHAKE algorithm was employed to constrain covalent bond lengths, including those involving hydrogen atoms (Miyamoto and Kollman, 1992). Temperature equilibration of the systems at 300 K was achieved via a Langevin thermostat, with a time step of 2.0 fs utilized for all the simulations. Minimization and equilibration protocols were executed for 10,000 steps and 10 ns, respectively. Subsequently, classical MD simulations lasting 100 ns, without constraints, were conducted for each protein‒ligand complex employing the Poisson‒Boltzmann (MM-PBSA) or generalized Born (MM-GBSA) and surface area continuum solvation approaches (Shityakov et al., 2017). Following the standard protocol, explicit solvation models were employed for all MD simulations, with implicit solvation (MM-PBSA/GBSA) utilized as a postprocessing end-state method to compute the binding free energies of molecules in solution, facilitated by a Python script (MM-PBSA.py).

## 3 Results and discussion

### 3.1 Experimental studies on the potential role of the hnRNP A2B1 protein in the radiomodulation of human endothelial cells

The expression of hnRNP A2B1 after irradiation was studied to establish its direct role in radiomodulation. We focused our research on human endotheliocytes because hnRNP A2B1 is pathogenetically significant both in the tumor parenchyma and its vascular stroma (endothelium). This year, we developed and patented a method for obtaining biopsy material of endothelium (endothelial cells) from excised hemorrhoidal nodes (HNs) [Patent RU 2022108179A, 28.09.2023, Bul. No. 28]. Experiments were conducted with the irradiation of human endothelial cells *in vitro* following the methodology described above. X-ray irradiation was performed at doses of 0.0 Gy, 0.5 Gy, 1.0 Gy, 2.0 Gy, 4.0 Gy, and 8.0 Gy. Twenty-four hours after irradiation and *in vitro* incubation, the cells were transferred to adhesive-coated slides and stained via an immunocytochemical method with the first monoclonal antibody against hnRNP A2B1.

We observed the following parameters in routinely stained preparations. Endothelial cells predominated in the cytograms (70%–80%). In the range from 0.0 Gy to 4.0 Gy, no significant shifts in the cytograms were observed. In samples irradiated with a dose of 8.0 Gy after 14 h, we observed a predominance of cells (up to 70%) with reduced sizes from 132.99 ± 20.31 µm (at 0.0 Gy) to 60.33 ± 6.38 µm (at 8 Gy). Simultaneously, we noted an increase in the frequency of anucleate CD31-positive cytoplasts and large (from 1 to 5 µm) extraendothelial vesicles from 3.5% without irradiation (0.0 Gy) to 7.8% after irradiation with 8.0 Gy.

**FIGURE 1:**
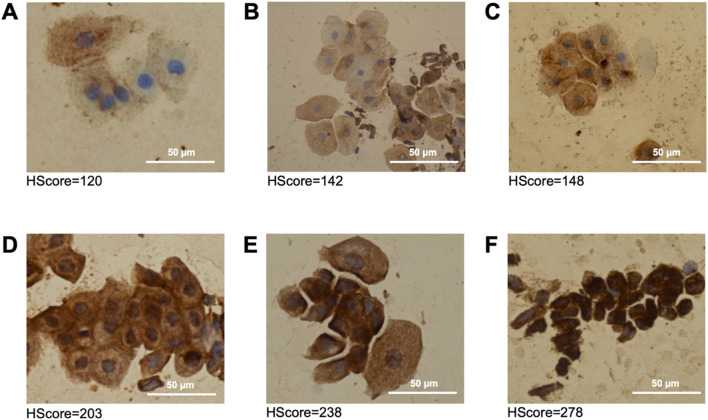
Immunocytochemical staining. Expression of the hnRNP A2B1 protein in human endothelial cells cultured in vitro for 24 h after X-ray irradiation at 0.0 Gy **(A)**, 0.5 Gy **(B)**, 1.0 Gy **(C)**, 2.0 **(D)** Gy, 4.0 Gy **(E)**, or 8.0 Gy **(F)**. Magnification 40x. Endothelial cells are indicated by black arrows.

Immunocytochemical staining of hnRNP A2B1 yielded the following results. In all the samples, including nonirradiated endothelial cells, we observed a positive immunocytochemical reaction in the cytoplasm, although small granules were also present in the nuclei. Histological sections of pathologically confirmed renal carcinoma served as the positive control for the immunocytochemical reaction, and for the negative control, we used smears of intact removed hemorrhoidal nodes. Importantly, hnRNP A2B1 is considered a “housekeeping” protein, so its background staining should be normal in all somatic cells. Figure 1 shows microphotographs of immunocytochemically stained endothelial cells. Most unexposed endothelial cells (0.0 Gy) were antigen negative (0) or weakly positive (+). Variants with high expression levels (+++) were not detected. The baseline S_H_ in unexposed endothelial cells was 105.

At a dose of 0.5 Gy, cells with high expression (+++) were also not detected, but the S_H_ increased to 142 points. A slight increase to an S_H_ of 149 points was recorded at a dose of 1.0 Gy. Endothelial cells with high hnRNP A2B1 expression (+++) appeared after irradiation at a dose of 2.0 Gy. At this dose, all 100% of the cells were antigen positive, indicating that they expressed hnRNP A2B1. At the 2.0 Gy dose, the S_H_ significantly increased to 204 points. After irradiation at a dose of 4.0 Gy, more than half of the cells presented high expression, and the S_H_ increased to 238 points. Finally, at a dose of 8.0 Gy, we observed very high staining intensity, with an S_H_ score of 280 out of a possible 300 points.

**FIGURE 2:**
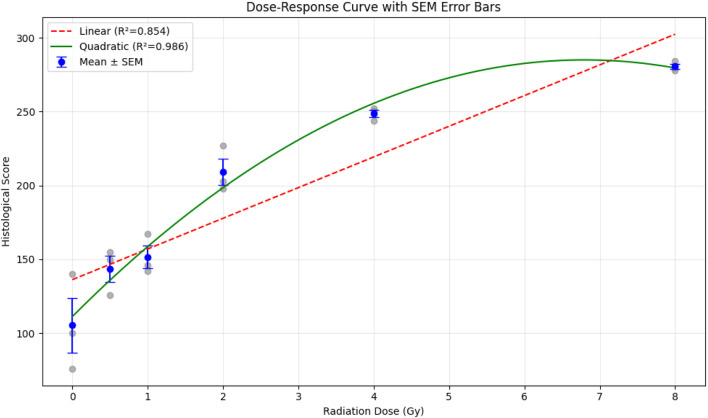
Dose‒response curve indicating hnRNP A2B1 expression in endothelial cells at different radiation dosages. The baseline hnRNP A2B1 expression thresholds for endothelial cells and human renal carcinoma are shown as dotted lines to ensure that the controlled experiments are rationalized. The statistics was calculated from three independent experiments. Error bars are explicitly defined as mean ± SEM.

The experimental data obtained are presented in Figure 2 as a hyperbolic/saturating relationship dose‒response curve.

The dose‒response dependence of hnRNP A2B1 expression in endothelial cells is described by the following equation:

$$S_{H}=\frac{578.7*DR}{6.86+DR}-17.16*DR+106.2$$

where Y represents the expression of hnRNP A2B1 (S_H_) and X represents the dose of X-ray irradiation (DR). The non-linear correlation coefficient (*R* ^2^) between the expression of hnRNP A2B1 and the irradiation dose was 0.99.

Since we used the standard and widely accepted method of assessing the results of immunohistochemical and immunocytochemical reactions via the S_H_, which is commonly used in clinical practice, we can make the following practical recommendations. The scoring ranges were as follows: 0 to 10, negative; 11 to 100, weakly positive; and 101 to 300, positive. For the clinical use, we suggest evaluating immunocytochemical reactions to hnRNP A2B1 as weakly positive up to 100 points as the background before irradiation, positive from 101 to 200 points, and strongly positive from 201 to 300 points as the S_H_. This is important because we anticipate the practical use of our findings to justify the prescription and monitoring of the effectiveness of radiomodulatory agents in oncology.

Statistical analysis of the dose-response data (n = 3 per dose) revealed a significant positive trend: mean scores increased from 105.3 ± 32.3 (95% CI: 25.0–185.7) at 0 Gy to 280.7 ± 3.1 (95% CI: 273.1–288.3) at 8 Gy. Linear regression showed a significant slope (20.8 points/Gy, 95% CI: 8.9–32.7, p = 0.008, *R* ^2^ = 0.854), but the data were better fit by a quadratic model (Score = 111.2 + 51.1 × Dose – 3.76 × Dose^2^, *R* ^2^ = 0.986). One-way ANOVA confirmed highly significant differences across doses (F(5,12) = 47.4, p = 1.7 × 10^−7^), with Tukey’s HSD post-hoc tests revealing robust separation from 2 Gy onward.

Thus, we investigated the potential role of the hnRNP A2B1 protein in the radioresistance of mammalian cells and concluded that the hnRNP A2B1 protein begins to be expressed in cells after irradiation. The role of the hnRNP A2B1 protein in cell radioresistance should now be considered evident rather than potential.

Since we established a dose-dependent hnRNP A2B1 expression parameter and substantiated the role of hnRNP A2B1 in radioresistance, the prospect of considering hnRNP A2B1 as a therapeutic target in malignant neoplasms arises. Notably, this target is pathogenically significant both in the tumor parenchyma and its vascular stroma (endothelium). We initiated the search for ligands that can selectively inactivate hnRNP A2B1.

### 3.2 Structure-based *in silico* screening

**FIGURE 3:**
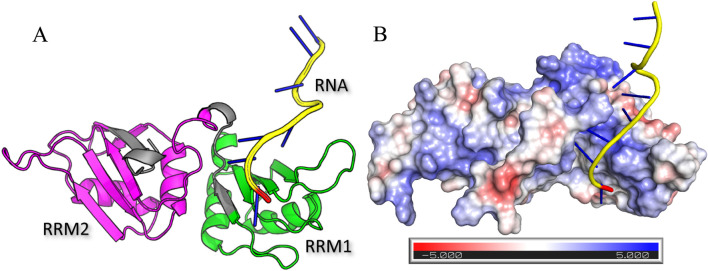
Crystal structure of hnRNP A2B1 in complex **(A)** with 10-dimensional RNA containing the RRM1 (green; positions: 19–98 aa) and RRM2 (magenta; 112–191 aa) RNA-binding domains and an RNA fragment (yellow) and electrostatic surface potential map **(B)** via the APBS algorithm.

The hnRNP A2B1 protein has several potential ligand-binding sites. In our study, two RNA-binding domains (RRM1 and RRM2) were used as binding pockets (Figure 3A). Figure 3B displays an electrostatic surface potential map of a protein‒RNA complex generated via the APBS algorithm. Protein‒RNA interactions are characterized by regions of positive charge on the protein surface, primarily due to the presence of basic amino acids such as lysine and arginine. These positively charged regions are crucial for binding to the negatively charged phosphate backbone of the RNA. This electrostatic complementarity enhances the stability and specificity of the protein‒RNA complex.

The rigid-flexible molecular docking of 33 ligands with radiomodulatory properties revealed that the binding energy of the interaction between hnRNP A2B1 and indralin was the lowest (−9.1 kcal·mol-1), and this affinity value was observed when the ligand interacted with the receptor in the RRM2 domain (Supplementary material, Supplementary Table S1). Additionally, among the natural compounds, hesperidin, which has a binding energy of −8.7 kcal·mol-1 in the RRM2 domain, and psoralidin, which has a binding energy of −9.0 kcal·mol-1 in the RRM1 domain, have the lowest total energies (Table 1).

**TABLE 1:** Best binding affinities represented as ΔG functions in kcal·mol^-1^ (top three protein-ligand complexes).

Compound	ΔG_bind_ (RRM1)	ΔG_bind_ (RRM2)	ΔG_tot_
Indralin	−7.6	−9.1	−16.7
Hesperidin	−8.5	−8.7	−17.2
Psoralidin	−9.0	−8.9	−17.9

**FIGURE 4:**
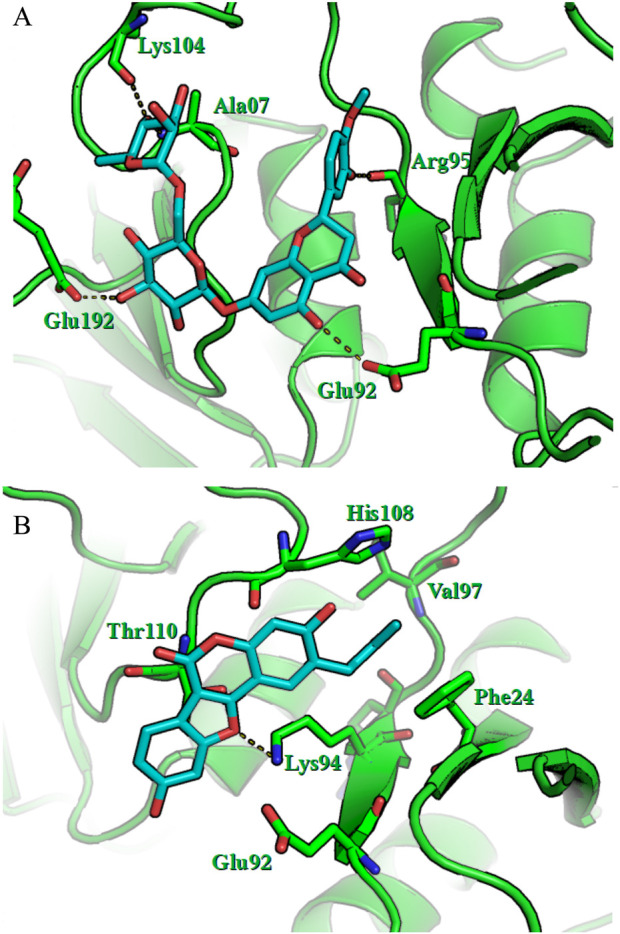
Visualization of the molecular docking of the hnRNP A2B1 protein with hesperidin **(A)** and psoralidin **(B)**. Green represents the hnRNP A2B1 protein, cyan represents ligands in the RRM1 or RRM2 pockets, and dashed lines represent hydrogen bonds.

Figure 4 shows the results of the molecular docking studies showing that hnRNP A2B1 interacts with the radiomodulator ligands hesperidin and psoralidin at the RRM1 position (19–98 aa). The RRM1 site was selected for further analysis because of its low protein‒ligand affinity. For hesperidin, the specific amino acids Lys104, Ala07, Arg95, Glu92, and Glu192 are labeled, indicating that the ligand fits within the protein binding pocket. Hydrogen bonds between hesperidin and the amino acids Lys104, Ala07, Arg95, Glu92, and Glu192 of the protein are depicted, revealing strong interactions that stabilize the protein‒ligand complex. For psoralidin, the highlighted amino acids His108, Val97, Phe24, Thr110, Lys94, and Glu92 have similar positions within the binding pocket. In particular, the hydrogen bond between psoralidin and Lys94 indicates a strong binding interaction. These observed interactions and the precise positioning of the ligands suggest that both hesperidin and psoralidin have significant affinities for hnRNP A2B1, underscoring their potential as effective radiomodulators.

**FIGURE 5:**
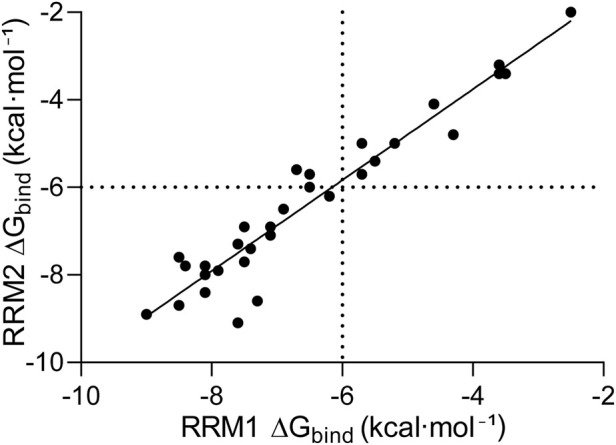
Correlations between the binding free energies of different ligands. The ligand binding sites RRM1 and RRM2 of hnRNP A2B1. The energy thresholds are depicted as dotted lines.

The results obtained from molecular docking were further analyzed via statistical methods (Figure 5). We found a significant correlation between ΔG_bind_ in RRM1 and ΔG_bind_ in RRM2 of hnRNP A2B1, with an *R* ^2^ of 0.92 (p-value <0.001), indicating that the amino acids in the binding site do not significantly impact the binding of a particular drug.

### 3.3 Molecular dynamics simulation

**FIGURE 6:**
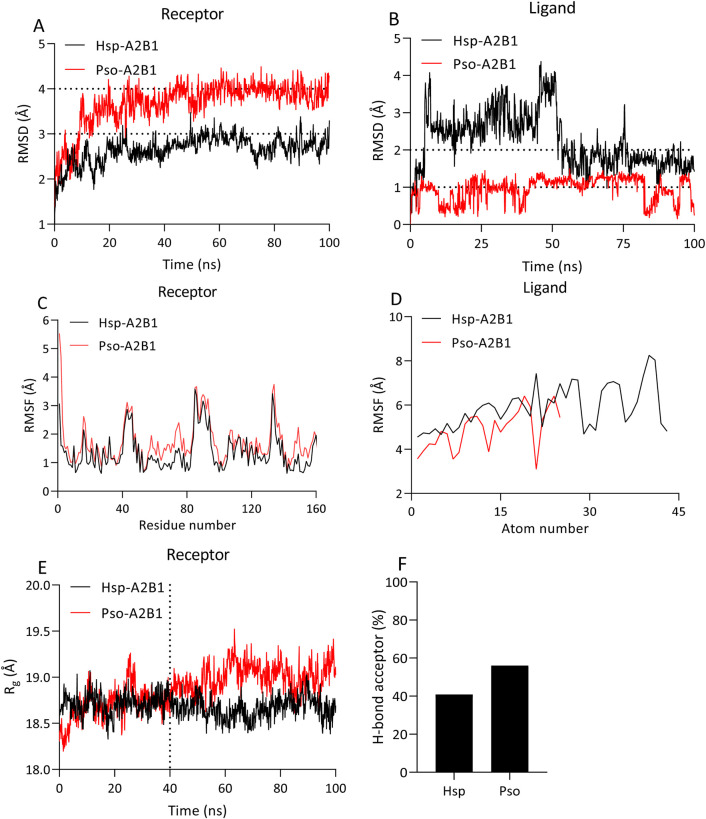
Analysis of the 100 ns MD trajectories of the protein‒ligand complexes. RMSD **(A,B)**, RMSF **(C,D)**, Rg **(E)**, and percentage of ligand H-bond acceptors **(F)** calculated for the Hsp-A2B1 and Pso-A2B1 complexes during a 100 ns MD simulation. The thresholds are depicted as dotted lines.

The best binders, hesperidin (Hsp) and psoralidin (Pso), to hnRNP A2B1 were selected for further molecular dynamics (MD) simulations to validate the results of previous molecular docking studies. We first calculated the root mean square deviation (RMSD) to measure structural variations between the initial and final structures of the protein‒ligand complexes. Compared with the Pso-A2B1 complex, which has an RMSD close to 4.0 Å, the Hsp-A2B1 complex has a lower RMSD, stabilizing at approximately 3.0 Å (Figure 6A), whereas the Pso-A2B1 system has a higher ligand RMSD, remaining within the 1.0 Å range, whereas the RMSD of the Hsp ligand deviated to approximately 2.0 Å after 100 ns (Figure 6B).

Root mean square fluctuation (RMSF) analysis was used to assess the flexibility of individual residues within the protein‒ligand complexes. The RMSF values exhibited similar patterns for both complexes, with elevated levels at the N- and C- termini and flexible secondary structure elements (loops). However, some residues in the Pso-A2B1 complex displayed slightly greater flexibility than did those in the Hsp-A2B1 complex (Figures 6C,D), indicating regions of more dynamic behavior in the Pso-A2B1 complex.

The radius of gyration (Rg) values, which measure the overall compactness of the protein during the simulation, indicated greater compactness for the Hsp-A2B1 complex than for the Pso-A2B1 complex. This suggests conformational changes in protein size and shape upon binding, which are more pronounced after the 40 ns time point (Figure 6E).

Analysis of the hydrogen bonds revealed a greater percentage of ligand H-bond acceptors in the Pso-A2B1 complex than in the Hsp-A2B1 complex (Figure 6F). This contributes to the enhanced binding stability observed in the Pso-A2B1 system, as stronger hydrogen bonding interactions are suggested by the greater percentage of hydrogen bond acceptors.

The binding affinities and associated energy terms derived from the 100 ns MD trajectories are summarized in Table 2. Notably, the generalized Born (ΔGgb) and Poisson‒Boltzmann (ΔGpb) solvation energies were more favorable for the Pso-A2B1 complex, indicating better solvation stability for this system. The analysis revealed that while both complexes exhibit strong gas‒phase binding energies, solvation energies play a crucial role in determining the overall binding affinity. The Pso-A2B1 complex, which has better solvation energy terms, emerges as a more stable complex under simulated conditions.

**TABLE 2:** Summary of binding affinities and associated energy terms, presented in kcal·mol^-1^. These values are derived from the analysis of 100 ns MD trajectories for the investigated protein‒ligand complexes.

Energies	Hsp-A2B1	Pso-A2B1
${\Delta G}_{gas}$	−58.86	−49.61
${\Delta G}_{solv}^{gb}$	47.68	21.19
${\Delta G}_{solv}^{pb}$	63.89	45.61
${\Delta G}_{gb}$	−11.18	−28.41
${\Delta G}_{pb}$	5.03	−4.57

Overall, the MD simulation results corroborate the molecular docking findings, confirming that the Pso-A2B1 complex has superior binding stability to that of the Hsp-A2B1 complex. This enhanced stability is attributed to stronger hydrogen bonding interactions and more favorable solvation energies, making Pso-A2B1 a potentially more effective candidate for further experimental validation and therapeutic development.

In molecular dynamics simulations, binding free energies for hnRNP A2B1 ligands are calculated using MM-GBSA and MM-PBSA methods. The gas-phase binding energy (ΔGgas) reflects van der Waals and electrostatic interactions, with negative values indicating stronger binding. Generalized Born ( ${\Delta G}_{solv}^{gb}$ ) and Poisson-Boltzmann ( ${\Delta G}_{solv}^{pb}$ ) solvation energies estimate polar solvation costs, where positive values denote desolvation penalties. Total binding free energies, ${\Delta G}_{gb}$ (GB model) and ${\Delta G}_{pb}$ (PB model), combine gas-phase, solvation, and non-polar terms, with more negative values signifying stronger affinity. All energies are in kcal·mol^-1^, derived from 100 ns MD trajectories.

The successful application of molecular docking can be seen in the following studies. Li et al. (2021) used docking to screen 4,621 approved drugs from DrugBank against the crystal structure of MAPK14 to identify a potential anti-inflammatory drug for treating chronic myeloid leukemia. The study identified a potent inhibitor, nilotinib, with an IC50 of 40 nM *in vitro*. Dakshanamurthy et al. (2012) demonstrated another successful application of computer modeling in drug discovery. In their methodology, the interaction of drug targets between 3,671 FDA-approved drugs and 2,335 human protein crystal structures was predicted with 91% accuracy. Additionally, Dakshanamurthy et al. reported that the antiparasitic drug mebendazole also possesses anticancer properties, exhibiting strong inhibitory activity against receptor 2 of vascular endothelial growth factor. The results were experimentally confirmed, showing that mebendazole effectively treats various models of medulloblastoma owing to its significant impact on tumor angiogenesis.

Through molecular docking, Dubovsky et al. demonstrated that ibrutinib, whose target is Bruton’s tyrosine kinase (BTK), has potential covalent binding to the interleukin-2–inducible kinase ITK at Cys442 and fills the active site, similar to the irreversible binding of ibrutinib to BTK. *In vitro* binding assays confirmed that ibrutinib could irreversibly bind a significant percentage of endogenous ITK in a T-cell leukemia cell line. A binding energy less than 0 indicates that ligands and receptors can bind spontaneously. A binding energy <−6.0 kcal⋅mol^-1^ indicates good binding activity (Shityakov and Forster, 2014; Kovalenko et al., 2023; Tamaian et al., 2023). The lower the binding energy is, the greater the binding activity, and the more easily the pharmacological agent binds to the receptor.

Chemical radiomodulators remain the subject of active research, as their effectiveness is often limited by high toxicity, side effects, and high cost. Owing to the toxicity of synthetic chemical compounds, interest in natural plants and phytochemicals as potential sources of radiomodulators has increased. Therefore, screening natural compounds is a major research direction for discovering new drugs.

In our study, we identified significant ligands with radiomodulatory properties that target hnRNP A2B1. These include indralin (B-190 (Vasin, 2021)), which has a binding energy of −9.1 kcal⋅mol^-1^ in the RRM2 region, and the natural compounds hesperidin and psoralidin, which have binding energies of −8.7 kcal⋅mol^-1^ and -9.0 kcal⋅mol^-1^ in the RRM2 and RRM1 regions, respectively (Table 1).

Epigallocatechin-3-gallate (EGCG), the primary polyphenolic component of green tea, is well known for its potent free radical scavenging properties. It has been shown to be effective in addressing a range of conditions, including antiaging, antiangiogenic, anti-inflammatory, and antiviral effects. Research indicates that EGCG inhibits oxidative stress through the Keap1/Nrf2 signaling pathway (Tang et al., 2024) and might also improve DNA repair following radiation, contributing to its radiomodulatory effects. Our findings further emphasize the pleiotropic nature of this compound.

To explore the functional relevance of hnRNP A2B1 in radiomodulation, we conducted preliminary micronucleus assays in U87MG and LN229 glioblastoma cells (unpublished data). In these experiments, hesperidin treatment administered 3 h after irradiation (2 Gy) reduced the frequency of radiation-induced micronuclei by approximately three-fold compared to irradiated controls, indicating a measurable radioprotective effect at the cytogenetic level. Although these preliminary observations require further validation in dedicated follow-up studies, they provide initial support for the hypothesis that modulation of hnRNP A2B1, via ligands such as hesperidin, can influence cellular responses to radiation.

## 4 Conclusion

In conclusion, this study underscores the critical importance of heterogeneous nuclear ribonucleoproteins (hnRNPs), particularly hnRNP A2B1, as promising targets for developing radiomodulatory drugs with radioprotective or radiosensitizing effects. Radiation modulation is crucial because of increasing radiation-related risks in the real world, and our research explored the potential of hnRNP A2B1 in this context. In vitro experiments on human endothelial cells exposed to various X-ray doses revealed a dose-dependent increase in hnRNP A2B1 expression, with the histochemical score increasing significantly from 105 to 280 at 8.0 Gy.

Using molecular docking, we evaluated 33 radiomodulatory agents for their binding affinities with hnRNP A2B1. Notable ligands, such as hesperidin and psoralidin, have strong binding affinities (ΔGbind values: −17.2 and −17.9 kcal mol^-1^). Molecular dynamics simulations further validated these interactions, with psoralidin demonstrating the highest affinity for hnRNP A2B1 via implicit solvation models.

These findings support the potential of hnRNP A2B1 as a therapeutic target for radiomodulatory drugs. By leveraging the principles of ergodicity (Shityakov et al., 2023), natural compounds such as hesperidin, which demonstrate radiomodulatory effects (Musa et al., 2019), might show promise for cancer treatment (Tang et al., 2023). Advancing theoretical modeling of radiomodulatory agents *in silico* could expedite and optimize the use of approved medicinal products, especially those of natural origin and devoid of side effects, for broad populations in emergency radiation scenarios. Ultimately, these insights pave the way for the development of effective radioprotective and radiosensitizing agents to improve radiation therapy outcomes and mitigate radiation-induced damage.

## Data Availability

The original contributions presented in the study are included in the article/Supplementary Material, further inquiries can be directed to the corresponding author.
