# Supplementary material for: hnRNP A2B1 as a promising therapeutic target for radiomodulatory drug development: evidence from computational and experimental studies
Source: Front Pharmacol. 2026 Feb 25;16:1704625. doi: 10.3389/fphar.2025.1704625 (PMC13006754; doi:10.3389/fphar.2025.1704625)
Supplement: Supplementary file 1 [file DataSheet1.pdf]

Table S1. Molecular Docking Results of Radiomodulating Ligands with hnRNP A2B1 Target at RRM1 and RRM2 Binding Sites. All ΔG units are present kcal·mol^{-1}.

Compound	ΔG_{RRM1}	ΔG_{RRM2}	ΔG_{tot} 5
Psoralidin	-9.0	-8.9	-17.9
Hesperidin	-8.5	-8.7	-17.2
Indralin	-7.6	-9.1	-16.7
Epigallocatechin-3-gallate	-8.1	-8.4	-16.5
δ-Tocotrienol	-8.4	-7.8	-16.2
Naphazoline	-8.5	-7.6	-16.1
Genistein	-8.1	-8.0	-16.1
Apigenin	-8.1	-7.8	-15.9
PAC5	-7.3	-8.6	-15.9
γ-Tocotrienol	-7.9	-7.9	-15.8
Chlorogenic Acid	-7.5	-7.7	-15.2
Tocopherol Succinate	-7.6	-7.3	-14.9
Curcumin	-7.4	-7.4	-14.8
Troxerutin	-7.5	-6.9	-14.4
Resveratrol	-7.1	-7.1	-14.2
N-Acetyltryptophan Glucoside	-7.1	-6.9	-14.0
Melatonin	-6.9	-6.5	-13.4
Ferulic Acid	-6.5	-6.0	-12.5
Coniferyl Aldehyde	-6.2	-6.2	-12.4
Zingerone	-6.2	-6.2	-12.4
Serotonin	-6.7	-5.6	-12.3
Mexamine	-6.5	-5.7	-12.2
Caffeine	-5.7	-5.7	-11.4
Sesamol	-5.5	-5.4	-10.9
Vanillin	-5.7	-5.0	-10.7
Zimosan A	-5.2	-5.0	-10.2
Diphtheria	-4.3	-4.8	-9.1
Amifostine	-4.6	-4.1	-8.7
AET	-3.6	-3.4	-7.0
Cystamine	-3.5	-3.4	-6.9
WR-1065	-3.6	-3.2	-6.8
Mercamine	-2.5	-2.0	-4.5
Lycopene	0.0	-4.1	-4.1

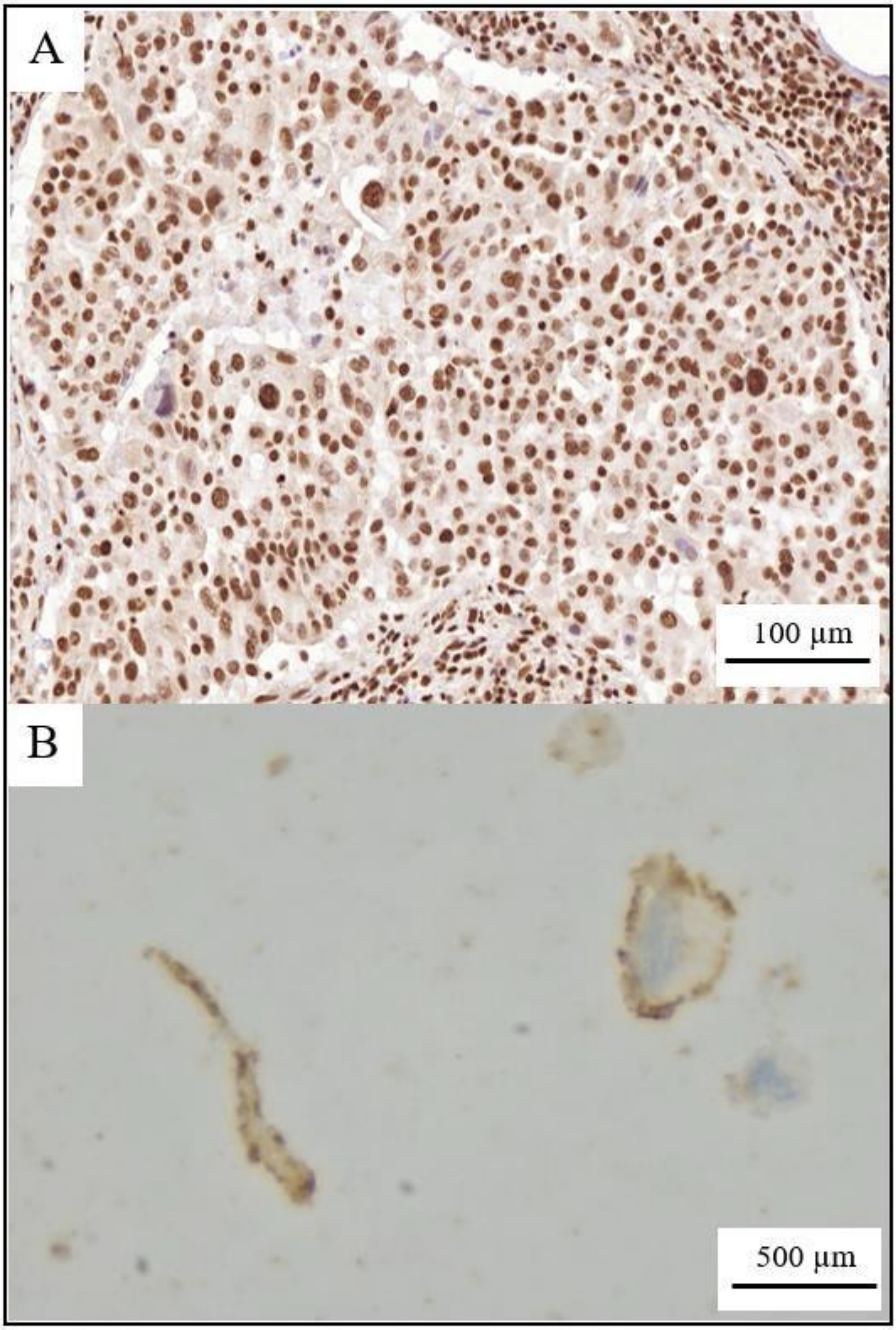

Figure S1. Expression of hnRNP A2B1 (brown color) in (A) renal carcinoma and (B) intact endothelial cells at 0.0 Gy being used as positive and negative controls.
